# Protocatechuic acid ameliorates neurocognitive functions impairment induced by chronic intermittent hypoxia

**DOI:** 10.1038/srep14507

**Published:** 2015-09-30

**Authors:** Xue Yin, Xiuli Zhang, Changjun Lv, Chunli Li, Yan Yu, Xiaozhi Wang, Fang Han

**Affiliations:** 1 School of Pharmaceutical Sciences, Binzhou Medical University, Yantai, Shandong, 264003, China; 2 Department of respiration, Binzhou Medical University Hospital, Binzhou, Shandong, 256603, China

## Abstract

Chronic intermittent hypoxia (CIH) is a serious consequence of obstructive sleep apnoea (OSA) and has deleterious effects on central neurons and neurocognitive functions. This study examined if protocatechuic acid (PCA) could improve learning and memory functions of rats exposed to CIH conditions and explore potential mechanisms. Neurocognitive functions were evaluated in male SD rats by step-through passive avoidance test and Morris water maze assay following exposure to CIH or room air conditions. Ultrastructure changes were investigated with transmission electron microscopy, and neuron apoptosis was confirmed by TUNEL assays. Ultrastructure changes were investigated with transmission electron microscope and neuron apoptosis was confirmed by TUNEL assays. The effects of PCA on oxidative stress, apoptosis, and brain IL-1β levels were investigated. Expression of Bcl-2, Bax, Cleaved Caspase-3, c-fos, SYN, BDNF and pro-BDNF were also studied along with JNK, P38 and ERK phosphorylation to elucidate the molecular mechanisms of PCA action. PCA was seen to enhance learning and memory ability, and alleviate oxidative stress, apoptosis and glial proliferation following CIH exposure in rats. In addition, PCA administration also decreased the level of IL-1β in brain and increased the expression of BDNF and SYN. We conclude that PCA administration will ameliorate CIH-induced cognitive dysfunctions.

Obstructive sleep apnea (OSA) when severe is characterized by repetitive upper airway obstruction during sleep, which leading to disruption of sleep and intermittent arterial oxygen desaturation^1^,^2^. It is a major public health problem and occurs in 4% of children and 5% of the general population^3^,^4^,^5^.

OSA is associated with a variety of cognitive deficits such as impairment in long-term memory, phonological, vigilance, decreased problem attention and irritability^6^,^7^,^8^,^9^,^10^,^11^. Chronic intermittent hypoxia (CIH) is a classical feature of OSA and is known to produce significant neurocognitive deficits, including spatial learning impairments in rats^9^. This evidence forms the rationale for linking chronic intermittent hypoxia to cognitive and behavioral dysfunction in patients with sleep apnea^12^. The patterns of cognitive disturbances in OSA patients are consistent with dysfunction of fronto-subcortical systems involved in executive and attention functions, such as the prefrontal cortex and the hippocampus^12^. Besides producing mechanistic insight, animal models can be used as platforms for testing preventive or restorative therapy.

Hypoxia mediated cognitive dysfunctions could due to neurotransmitter synthesis and release and altered protein expression^13^,^14^. A number of studies have revealed impairments in synaptic plasticity, occurrence of neuronal cell apoptosis, glial proliferation and brain-derived neurotrophic factor (BDNF) expression in intermittent hypoxia conditions, all of which might contribute to the neurocognitive impairment^3^,^15^,^16^,^17^. CIH-mediated cortical neuronal apoptosis and neurocognitive dysfunction maybe partially attributed to increased reactive oxygen species (ROS) production and oxidative stress propagation^16^. Studies have shown that increased activation of NADPH oxidase occurs and possibly underlies subsequent CIH-induced carbonylation of proteins, which activate microglial proinflammatory responses and promote such positive feedback loop^18^. In addition, a stimulation of interleukin-1β (IL-1β) would increase the level of SAPK/JNK phosphorylation and lead to an impairment of long-term synaptic plasticity^19^. There may be a role for BDNF, a member of the neurotrophin family that plays a role in neuronal survival and differentiation^20^,^21^,^22^. In the presence of intermittent hypoxia, a significant reduction in the expression of BDNF occurs, an action which may not only contribute to a failure to prevent neuronal injury, including apoptosis, induced by ROS, but also to an impairment in long-term synaptic plasticity^23^. There was a significant reduction in the expression of BDNF which possibly not only contributes to fails to prevent neuronal injury, including apoptosis, induced by ROS but also impaired long-term synaptic plasticity^23^.

Protocatechuic acid (3, 4-dihydroxybenzoic acid, PCA), a simple phenolic compound, is plentiful in edible fruits and vegetables and also naturally present in many folk medicine such as Hibiscus sabdariffa^24^ and Salvia miltiorrhiza^25^. PCA has antioxidant, anti-aging, anti-inflammatory, and anti-atherosclerotic activities, suggesting neurological protective properties^26^. PCA will lower the expression of cleaved caspase-3 and decrease the rate of cardiomyocyte apoptosis induced by hypoxia/reoxygenation^27^. Recently, PCA was proven to have the neuroprotective effects in PC12 cells and *in vivo* in rats in part by inhibiting free radical generation and promoting endogenous antioxidant enzymatic activities^28^,^29^. On account of these potential effects, there is a need to examine whether PCA has neuroprotective effects in rats exposed to CIH, and, if true, indicate mechanisms for its action.

There is a hypothesis that PCA has neuroprotective effects in CIH rats through by inhibiting apoptosis, glial proliferation and associate with synaptic plasticity. Morris water maze for acquisition and retrieval and the step-through passive avoidance test were performed to evaluate behavioral performance in our study. The effect of PCA administration on apoptosis was investigated. Furthermore oxidative stress parameters which are related with apoptosis were studied. The expression of glial fibrillary acidic protein (GFAP) and synaptophysin (SYN) that are respectively associated to glial proliferation and synaptic plasticity were investigated. We also measured the level of IL-1β, expression of BDNF and JNK, P38, ERK to find other potential mechanism of the PCA maybe in occurrence of neuronal apoptosis and impairments of synaptic plasticity.

## Result

### Protocatechuic acid protects CIH rats’ learning and memory

**Figure 1:**
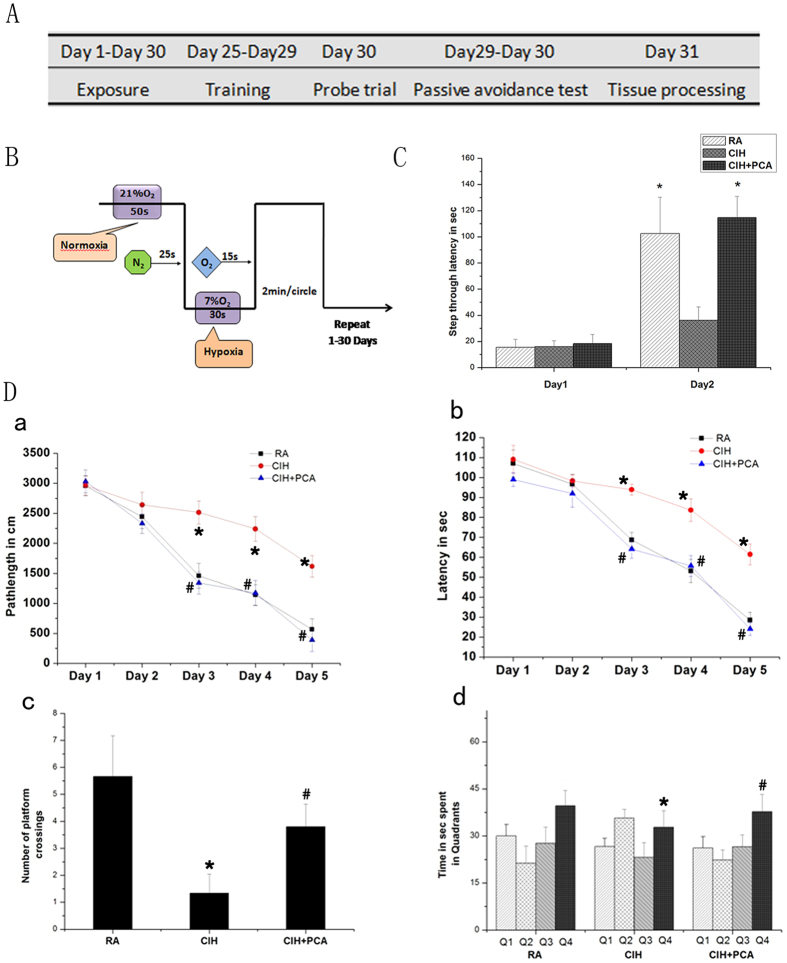
(**A**,**B**) Chronic intermittent hypoxia protocol. (**C**) Step-through passive avoidance test. We measured latency until rats first entered into the dark chamber. *P < 0.05 indicates a significant difference from the CIH group.(**D**, a) Pathlength of rats during training in Morris water maze. PCA administration decreases the pathlength in rats trained in Morris water maze. (**D**, b) Latency of rats during training in Morris water maze. PCA administration deceases the latency. (**D**, c) Number of times rats cross the platform position during probe trail. (**D**, d) Time. spent in the quadrants during probe trail. The fourth quadrant (Q4) of the Morris water maze had the submerged platform. All data points are mean ± SD. * denotes p < 0.05 when compared to RA group and # denotes p < 0.05 when compared to CIH group. n = 15.

In step-through passive avoidance test, latency time, relative to the motor and explorative activity of rats, showed no statistically significant differences among groups, demonstrating that animals behave the same during the acquisition trial (Fig. 1C). In the retention trial, a change in step-through latency time indicates the consolidation of the reinforced stimuli in the memory of rats. CIH significantly decreased latency compared to the RA group (36.1 ± 10.3 vs. 102.5 ± 27.9, p < 0.05). On the contrary, PCA treated rats showed significantly higher latency compared to the CIH group (114.7 ± 16.1 vs. 36.1 ± 10.3, p < 0.05) on day 2. Briefly, PCA exhibited significant protective effects on learning/memory ability of rats compared to the CIH groups (Fig. 1C).

In Morris water maze training, rats exposed to CIH showed increased latency period and pathlength on days 3, 4 and 5 of training when compared to the RA group. While rats in Group III showed a significantly lower latency and pathlength on the same days as compared to the animals in CIH group (p < 0.05, Fig. 1D a,b). The results of probe trail revealed a decrease in preference for the target quadrant in CIH rats when compared to the RA rats (32.8 ± 5.2 vs. 39.6 ± 4.9, p < 0.05). Rats in Group III spent maximum time in the target quadrant compared to the CIH treated rats (37.8 ± 5.4 vs. 32.8 ± 5.2, p < 0.05, Fig. 1D c). The mean number of platform crossings in the CIH treated rats was lowest while that of the PCA treated rats was nearly the same as that of RA rats (Fig. 1D d).

### The cognitive deficit stimulated by CIH is associated with apoptosis in hippocampal and prefrontal cortex and PCA inhibits the apoptosis

**Figure 2:**
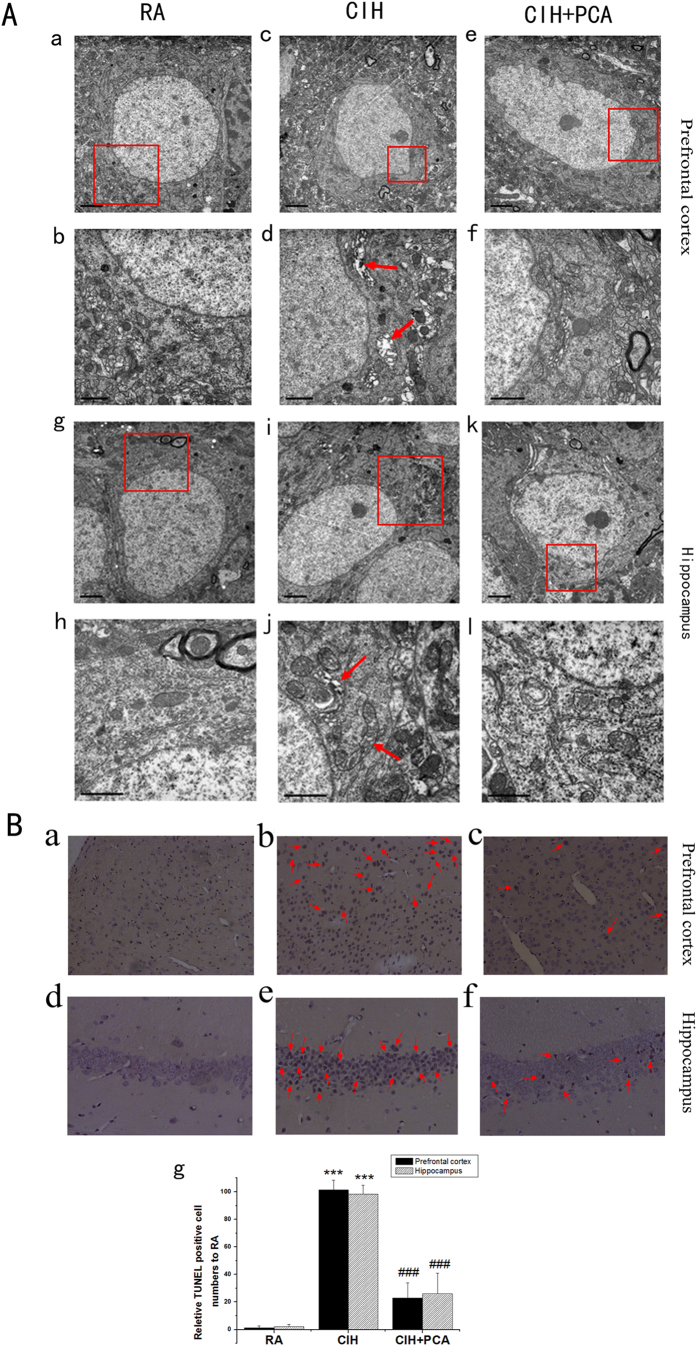
(**A**) Ultrastructural changes by transmission electron microscopy. No abnormal ultrastructural changes were found in the neurons of the prefrontal cortex (a,b) and hippocampus (g,h) in RA group. Karyopyknosis, chromatin margination and karyotheca breakage, mitochondria abnormalities including swelling, vacuolation and breakage of the cristae, Golgi body and endoplasmic reticulum swelling in the cortex of the prefrontal lobe (c,d) and hippocampus (I,j) were evident in the CIH treated groups. In PCA treated group, the pathological changes in the neurons of the prefrontal cortex (e,f) and hippocampus (k,l) were improved remarkably compared to CIH groups. Scale bar is 2 μm. (**B**) TUNEL staining for apoptotic neurons in the prefrontal cortex (a–c) and hippocampus (d–f) of each group (original magnification ×400). TUNEL positive neurons were stained in brown and the mean was calculated from five sections. Percentages of neurons apoptosis was shown from rats exposed to RA or CIH and treated with PCA (g). The result is reported as means ± SD, **** denotes P < 0.001 when compared to RA group and ### denotes p < 0.001 when compared to CIH group. n = 3.

To assess the neuronal injury in the relevant brain regions of spatial memory, we evaluated the ultrastructural changes in preprefrontal cortex and hippocampus with electron microscope. There were no obvious abnormal ultrastructure changes in the neurons of the prefrontal cortex (Fig. 2A a,b) and hippocampal (Fig. 2A g,h) in the RA group. However, in the CIH exposed group, there was a series of changes including karyopyknosis, chromatin margination and karyotheca breakage in the prefrontal cortex (Fig. 2A c,d) and the hippocampus (Fig. 2A I,j). We observed mitochondria abnormalities including swelling, vacuolation and breakage of the cristae, Golgi body and endoplasmic reticulum swelling in the prefrontal cortex (Fig. 2A c,d) and the hippocampus (Fig. 2A i,j). In PCA treatment group, the pathological changes were improved remarkably compared to CIH groups (Fig. 2A e,f,k,l).

To corroborate these results, apoptosis was also evaluated by TUNEL assay. TUNEL positive cells spread in the hippocampal CA1 and cortex of the prefrontal lobe of CIH groups were shown in Fig. 2B. Percentage of TUNEL positive cells in the hippocampus and the prefrontal cortex in PCA treated rats reduced as shown in Fig. 2B compared with CIH group. These results confirmed occurrence of neuronal cell apoptosis in CIH group and antiapoptotic effect of PCA.

**Figure 3:**
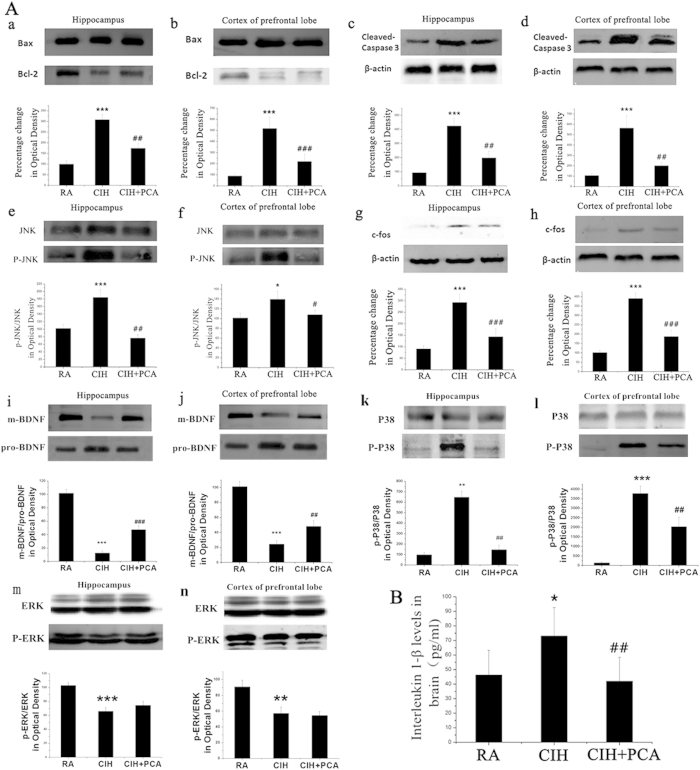
(**A**) JNK and P38 pathways are active and apoptosis is triggered after CIH treatment. PCA treatment inhibited the response. Representative Western blotting images of Bcl-2 (a,b), Bax (a,b), Cleaved-Caspase3 (c,d), phosphor-JNK (e,f), JNK (e,f), c-fos (g,h), BDNF (i,j), pro-BDNF (i,j), phosphor-P38 (k,l), P38 (k,l) phosphor-ERK (m,n), ERK (m,n) expression in hippocampus and prefrontal cortex after CIH and (or) PCA treatment. Full-length blots/gels are presented in Supplementary Figure A and B (n = 3 rats in each group). (**B**) Effects of PCA on the brain levels of Il-1β in the CIH-induced rats. *P < 0.05, **P < 0.01, ***P < 0.001 vs. RA rats; #P < 0.05, ##P < 0.01, ###P < 0.001 vs. CIH rats. n = 8.

Bax, Bcl-2 and Cleaved Caspase-3 expression were also used to study and confirm apoptotic cell death in rat hippocampus and the cortex of prefrontal lobe following exposure of rats to CIH. Activation of Caspase activity contributes to apoptotic cell death in many systems including central nervous system^30^. The Bcl-2 family of proteins is related to positive and negative regulation of apoptosis^31^. Both in hippocampus and prefrontal cortex, the ratio of Bax/Bcl-2 increased in CIH treatment rats as compared to control group (Fig. 3A a,b). In camparison, the ratio of Bax/Bcl-2 decreased in PCA treated group (Fig. 3A a,b) when compared with CIH group. It was shown that CIH significantly increased the expression of Cleaved Caspase-3 (Fig. 3A) compared with RA group, by contrast, with PCA treatment there was increased Cleaved Caspase-3 expression.

### Apoptosis is associated with increased JNK and P38 phosphorylation in CIH exposure rats and PCA prevents JNK and P38 phosphorylation

To elucidate mechanisms underlying the increase in cell apoptosis with CIH exposure, several key components of the MAPK pathway were analyzed by Western blotting. In line with the increased Cleaved Caspase-3 in the samples, JNK and P38 phosphorylation levels and expression of c-fos were significantly increased in CIH animals compared to control rats (Fig. 3A e–h,k,l). JNK and P38 phosphorylation levels in PCA treated rats were decreased compared with the CIH group. A reduction of c-fos expression was also observed in PCA treated rats. The phosphorylation levels of ERK were decreased in CIH animals compared to control rats (Fig. 3A m,n). However, there was no significant difference between CIH and CIH+PCA group in phosphorylation levels of ERK. Together, these results suggest that the occurrence of neural cell apoptosis maybe in relation to P38/JNK-mediated pathways and PCA could inhibit the apoptosis of nerve cells hippocampus and the cortex of the prefrontal lobe, and by analogy mitigate behavioral changes produced by CIH, through these specific pathways.

### Effects of PCA treatment on interleukin 1β in CIH rat hippocampal and prefrontal cortex

In order to investigate whether the interleukin participation in the therapeutic effect of PCA to CIH rats, we examined the levels of Interleukin 1-β in brain tissues. The levels of Interleukin 1-β in CIH rats brain were increased significantly compared to control rats as shown in Fig. 3B (46.28 ± 17% vs. 73.1 ± 19.48%, n = 8) . In addition, rats administered with PCA under the same CIH conditions showed a significant decrement of interleukin 1-β levels (73.1 ± 19.48% vs. 42.04 ± 16.36%, n = 8).

### Activities of antioxidant enzyme, oxidase and MDA content

**Figure 4:**
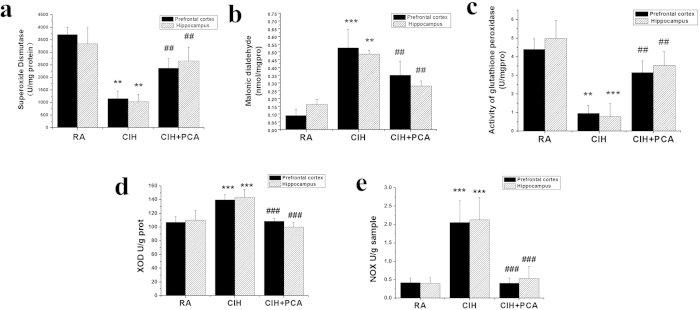
Effects of PCA on brain levels of SOD (**a**), MDA (**b**), GSH-Px (**c**), XOD(**d**) and NOX (**e**) against CIH-induced damage in rats. Values are presented as means ± SD for 10 mice in each group. *p < 0.05, **p < 0.01 and ***P < 0.001, vs. the RA group. #p < 0.05, ##p < 0.01 and ###P < 0.001, compared to CIH group. n = 8.

The activities of GSH-Px and SOD were significantly decreased in both the prefrontal cortex and hippocampus in the CIH group compared with the control group as shown in Fig. 4 a,c. On the other hand, under the same CIH conditions, rats administered with PCA showed a significant recovery of GSH-Px and SOD activities. The MDA contents in both the prefrontal cortex and hippocampus in the CIH group were increased compared with the control group as shown in Fig. 4b while the concentrations of MDA in CIH+PCA group were declined compared with CIH group. The activities of XOD and NOX were significantly elevated in both the prefrontal cortex and hippocampus in the CIH group compared with the control group as shown in Fig. 4d,e. However, rats administered with PCA showed a significant decline of XOD and NOX activities. In addition, PCA reduced the activity of XOD and NOX and the concentrations of MDA while increased the activity of GSH-Px and SOD. This observation indicates that PCA is capable of attenuating IH-induced increases in oxidative stress.

### Effects of PCA treatment on synaptic plasticity in CIH rat hippocampal and prefrontal cortex

Having shown that apoptosis in the brain tissues is associated with increased SAPK/JNK phosphorylation, and knowing that synaptic plasticity is closely related to memory, we explored whether SAPK/JNK phosphorylation may increase synaptic plasticity. Thus, the expression of SYN in brain tissues was analyzed using biochemistry techniques.

**Figure 5:**
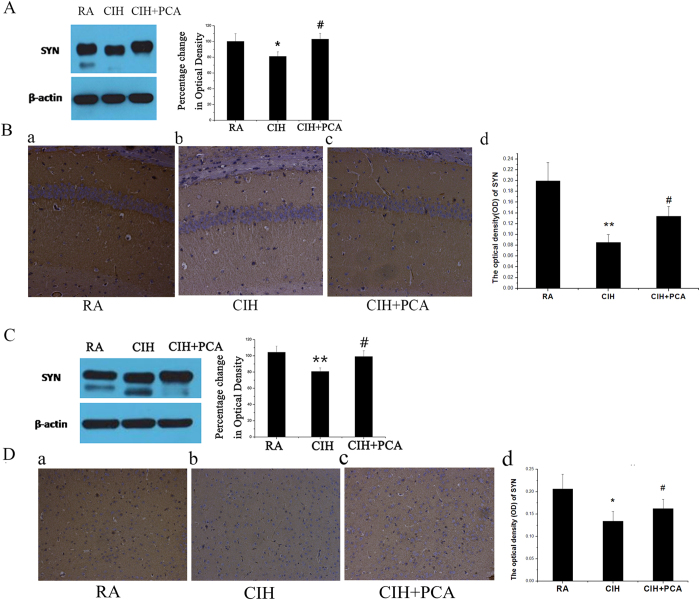
(**A**) Western blotting analysis revealed SYN protein bands of hippocampus. (**B**) SYN positive products (a–c) were brownish yellow granules located in the neuropil. Scale bar = 50 μm. The OD of SYN was shown in a statistical graph (d). The results were expressed as the mean ± SD (n = 3). **P < 0.05 vs. RA group, #P < 0.05 vs. CIH group. (**C**) Western blotting analysis revealed SYN protein bands of prefrontal cortex. (**D**) SYN positive products (a–c) were brownish yellow granules located in the neuropil. Scale bar = 50 μm. The OD of SYN was shown in a statistical graph (d). The datas were expressed as the mean ± SD (n = 3). *p < 0.05, **p < 0.01 and ***P < 0.001, vs. the RA group. #p < 0.05, ##p < 0.01 and ###P < 0.001, compared to CIH group. Full-length blots/gels are presented in Supplementary Figure A.

SYN expression in the CIH group was significantly lower compared to RA group. The SYN expression in PCA treated rats is high, and its IOD values were significantly higher compared to CIH group both in hippocampus and prefrontal cortex (Fig. 5A,C). Immunohistochemical (IHC) staining for SYN showed brownish yellow granules that were located in the neuropil, but not in the nucleus and perikaryon (Fig. 5B a–c). In addition, the staining was located in neuropil of prefrontal cortex (Fig. 5D a–c). The expression of the SYN protein in hippocampus and prefrontal cortex by IHC method was consistent with the SYN protein bands detected by WB among the different groups. The OD values of hippocampus were 0.199 ± 0.034, 0.085 ± 0.014 and 0.134 ± 0.019, respectively, in the RA group, CIH group and CIH+PCA group (Fig. 5B d). The OD values of prefrontal cortex were 0.201 ± 0.021, 0.142 ± 0.014 and 0.174 ± 0.025 (Fig. 5D d). Results demonstrate that CIH results in a down-regulation of the protein levels of SYN, and PCA treatment significantly increases the level of SYN protein in hippocampus (Fig. 5B d) and prefrontal cortex (Fig. 5D d).

### PCA treatment restores BDNF/pro-BDNF expression in CIH rat hippocampal and prefrontal cortex

As is known that both the dimer and monomer forms of BDNF are reduced after CIH treatment, we examined whether PCA treatment restores BDNF expression, and BDNF/pro-BDNF was analyzed by Western blotting. It was shown that BDNF/pro-BDNF is increased by half in CIH+PCA group compared with CIH rats both in hippocampus (Fig. 3A i) and the cortex of the prefrontal lobe (Fig. 3A j). The augmentation of BDNF/pro-BDNF of hippocampal and prefrontal cortex in PCA treatment group appears, at least in part, to result in the decreased apoptosis of neurons and the increased expression of synaptic plasticity.

### PCA administration attenuated CIH-induced glial proliferation in prefrontal cortical and hippocampus

**Figure 6:**
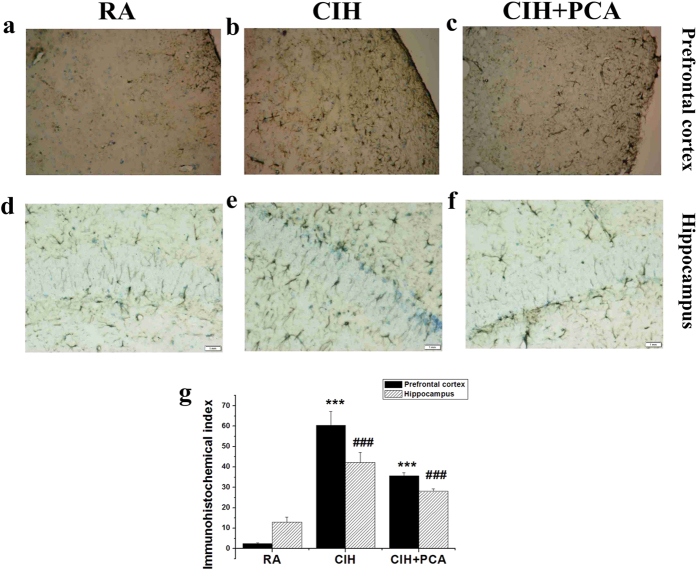
IHC index in rats exposed to room air (RA) or intermittent hypoxia (IH) and treated with PCA (***P < 0.001 vs. RA rats; P < 0.001 vs. CIH rats ; n = 3 rats in each group).

Immunohistochemistry (IHC) was performed for the astrocyte marker GFAP in order to determine whether PCA can affect glial proliferation after CIH exposure (Fig. 6). The IHC index of hippocampus was 2.508 ± 0.363, 60.472 ± 6.685 and 35.76 ± 1.419, respectively, in the RA group, CIH group and CIH+PCA group. The OD values of prefrontal cortex were 13.022 ± 2.429, 42.290 ± 4.736 and 28.122 ± 1.263. PCA administration reduced expression of GFAP in the cortex and hippocampus, indicating that our discovery provided further support for the view that PCA administration was able to attenuate CIH-induced increases in glial proliferation under CIH conditions.

## Discussion

An optimal animal model to copy the clinical and pathological features of OSA has not been well established. Most rat models of sleep-disordered breathing incorporate episodic hypoxia profiles consisting of alternating room air and 10% oxygen either every 90 sec or every 30 min during sleep^9^. We chose to model the effects of moderately severe OSA with the O_2_ concentration changed from 7.0 ± 1.5% to 21.0 ± 0.5% every 2 minutes for 8 hours during the rat diurnal sleep period for 30 days. The model made possible further study of the underlying mechanisms of CIH-associated neurocognitive deficits.

Consistent with previous reports, memory impairment was induced by intermittent hypoxia, as indicated by decreased time spent in the target quadrant and number of platform crossings as well as the increase in the pathlength and latency^32^. However, animals in group CIH+PCA performed better in the water maze, showing the lower latency and path length than the CIH rats, and significantly longer time spent in target quadrant and higher number of platform crossings as compared the CIH rats. These results indicate that administration of PCA could ameliorate learning and memory impairment induced by chronic intermittent hypoxia.

The hippocampus and prefrontal cortex, critical structures for learning and memory, are particularly sensitive to the hypoxic events occurring during extended periods of episodic hypoxia during sleep, and these changes lead to significant cognitive deficits in the rodent^33^,^34^. Consistent to previous researchers’ results, apoptosis of neuronal cells in the hippocampal and prefrontal cortex areas was found in CIH rats by TUNEL assays in our research. Besides, ultrastructure damages of neuronal cells in the hippocampus and prefrontal cortex in CIH group were found with transmission electron microscopy. The ratio of Bax/Bcl-2 and Cleaved Caspase-3 expression also increased in CIH rats in comparison with RA animals by Western blotting, further verified apoptosis occurred in CIH rats.

Increasing evidence suggests that the adverse neurobehavioral consequences imposed by intermittent hypoxia, at least in part, is caused by activation of oxidative stress as well as inflammatory signaling cascades^3^. In addition, several recent reports indicated increased oxidative stress plays a central role in mediating cell death in hypoxia^35^. Though several studies on PCA have demonstrated its antioxidant properties, anti-inflammatory activity and neurological protective effects^26^, not much is known on the action of the drug on molecular mechanisms. The effect on PCA administration on resisting apoptosis, an action crucial to memory consolidation, was measured by examining the ratio of Bax/Bcl-2, phosphor-JNK/JNK, phosphor-P38/P38, phosphor-ERK/ERK and the expression of c-fos and Cleaved Caspase-3. The activities of SOD, GSH-Px, XOD, NOX and contents of MDA were also studied.

SOD and GSH-Px, as endogenous antioxidant enzymes, are known to constitute a mutually supportive team of defense against reactive oxygen species (ROS)^36^. We also observed that PCA administration attenuated the decreased activities of SOD and GSH-Px induced by CIH. MDA, as one of the most abundant lipid peroxidation, is a commonly used indicator of oxidative stress. In our study, we observed that CIH was associated with significant increases in MDA in the hippocampus and prefrontal cortex, and the level was decreased in animals receiving PCA treatment. Studies have shown that intermittent hypoxia increases NADPH oxidase subunit protein expression in hypoxia sensitive brain regions involved in learning and memory and that PCA administration was able to attenuate the increase in NADPH oxidase activity after CIH exposure. Xanthine oxidoreductase system is a major source of cellular ROS, which contains XOD catalyzing the oxidation of purines and generating superoxide radicals as by product^37^. PCA administration also attenuated the rise of XOD activity.

The ratio of Bax/Bcl-2 and expression of Cleaved Caspase-3 were decreased in rats treated with PCA in comparison with CIH exposure rats. The results suggested there is an increased oxidative stress in rat brain hippocampal and prefrontal cortical neuronal cells. The increased oxidative stress during the reoxygenation phase results in increased expression and activation of caspase-3 and leads to neuron apoptosis. Therefore, consistent with previous observations, an antioxidant treatment could improve cell viability during oxidative stress induced by hypoxia-reoxygenation injury^38^.

Previous studies have confirmed that CIH can cause the JNK and P38 phosphorylation, thereby inducing neuronal cell apoptosis. In our study CIH treatment enhanced JNK and P38 phosphorylation, but phosphorylation levels were significantly decreased in PCA treatment rats compared to CIH group. We consider that PCA can inhibit phosphorylation of JNK and P38 against apoptosis. The phosphorylation levels of ERK were decreased in CIH animals but PCA has little influence on it. So we hypothesized that PCA mainly through influence JNK and P38 MAPK signal transduction pathways. Whether the PCA can protect cell from apoptosis through other way, we need further research.

There is evidence that BDNF can significantly prevent neuronal damage caused by oxidative stress, as found in neurodegenerative diseases^39^. Previous research has also found that it is possible that lack of BDNF in intermittent hypoxia fails to prevent apoptosis^3^. Association was confirmed in our experiment in that BDNF expression in CIH group decreased as compared to RA group by Western blotting. Most importantly, our studies confirmed PCA administration to significantly increase the level of BDNF expression during a period of CIH, in the hippocampus and prefrontal cortex. Therefore, PCA is likely to protect nerve cells against apoptosis by increasing BDNF expression.

Most importantly, our studies also confirmed PCA administration significantly increased the level of BDNF expression during the period of CIH in the hippocampus and prefrontal cortex. Therefore, PCA is likely to protect nerve cells against apoptosis by increasing BDNF expression.

Intermittent hypoxia impairs long-term potentiation (LTP), a neuronal signature for learning and memory^15^. Specifically, prior art has demonstrated that 7-day and 14-day CIH treatments could impair LTP in the CA1 region of the mouse hippocampus^3^. Consistent with this literature, a down-regulation of SYN expression was observed in hippocampal and prefrontal cortex of CIH treated Sprague Dawley rats in our experiment. JNK pathway activation has been shown to mediate impairment of hippocampal LTP by IL-1β^19^. Given the important role of IL-1β in mediating impairment of synaptic plasticity, we measured IL-1β levels. Consistent with previous findings^19^, IL-1β increased significantly in CIH treated animals. We therefore suspect that the impairment of synaptic plasticity in the hippocampal CA1 region may be associated with JNK phosphorylation mediated by IL-1β. To determine whether the neuroprotective properties of PCA treatment were responsible for the activity of IL-1β/JNK/LTP pathway, the next experiment was conducted via detecting the expression of SYN. We observed the SYN expression increased in CIH+PCA group in comparison with CIH, which may be due to the result that PCA decreased IL-1β and JNK phosphorylation. It was shown by others that decreased expression of BDNF was associated with impaired LTP induced by CIH treatments in the CA1 region of the mouse hippocampus^3^. Therefore, an increase in BDNF expression in the PCA treated CIH rat probably contributed to improved long-term synaptic plasticity in the hippocampus.

CIH group rats showed an increased IL-1β but it was significantly decreased in the CIH + PCA group. An inflammation reaction was observed in CIH rat brain. Glial proliferation is closely related to the inflammatory response. Reactive gliosis is a key component of the cellular response to central nervous system injury and comprises several changes in astrocytes and microglia. Angelo *et al.* have demonstrated that increased glial proliferation occurs in the cortex of rats exposed to intermittent hypoxia. The proinflammatory RAGE/NF-κB pathway is involved in neuronal damage and reactive gliosis in a model of sleep apnea by intermittent hypoxia^17^. Our findings were consistent with the actions of another polyphenol, Green tea catechin polyphenols. This compound could not only act to prevent oxidative stress but also attenuate reactive gliosis in the rat cortex and hippicampus exposed to IH^18^.

Our data provide evidence of a significant neuroprotective effect of PCA in CIH rats. PCA administration inhibited apoptosis by decreasing JNK/P38 phosphoryltion and the downstream target including c-fos, Bax and Cleaved Caspase-3 in the hippocampal and prefrontal cortex areas. Furthermore, SYN expression was decreased in CIH rat hippocampal area and these effects were reversed by PCA. Compared with CIH treated animals, PCA administration increased BDNF expression. Our results indicated that PCA offered neuroprotection against CIH induced neuron apoptosis and synaptic plasticity deficits through upregulating BDNF expression and inactivating the JNK/P38 signaling cascade. In addition, although the glial proliferation observed in our model is a reactive response, by-products of oxidative stress, PCA may conserve cognitive function by modulating cell glial proliferation.

**Figure 7:**
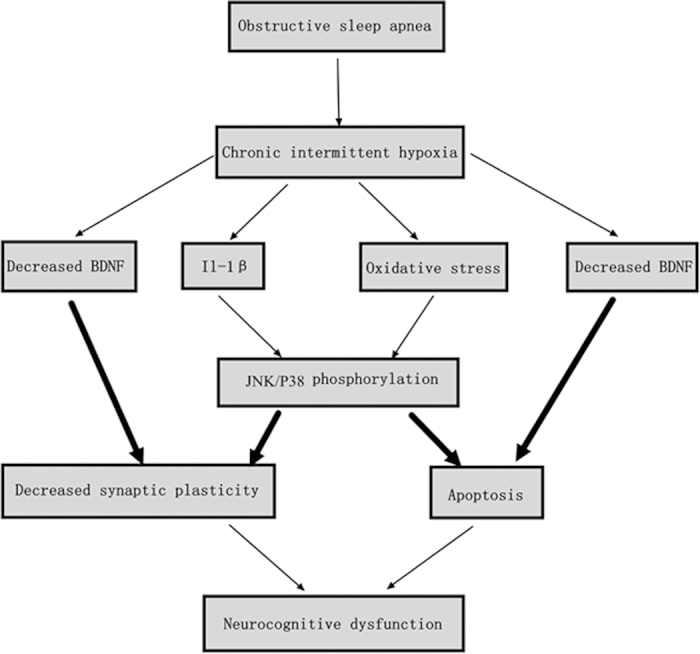
Potential mechanisms of PCA in ameliorating the neurocognitive dysfunction induced on exposure to CIH in a rat model of OSA.

This preclinical study explored the potential of PCA as therapy to prevent a morbidity of memory impairment induced by exposure to hypoxia/reoxygenation cycles that model the consequences of OSA (Fig. 7). PCA appears to influence CIH brain responses and protect from apoptosis, and favorably modulate glial proliferation and synaptic plasticity. Furthermore, PCA enhanced BDNF expression to improve long-term synaptic plasticity in the hippocampus and inhibit brain apoptosis. In addition, the present research also confirmed the antioxidant potential of PCA, therefore establishing it as effective therapeutic agent for clinical treatment of many neurological diseases associated with oxidative stress.

## Materials and Methods

### Animals

Male Sprague Dawley rats weighing 220 ± 15 g and 3 months of age were used. The animals were group housed in clean polypropylene cages with day and night cycle of 12 h each. The temperature was maintained at 22 ± 2 °C at 65 ± 5% humidity. Food and water were provided ad libitum. The experimental protocol was approved by the ethics committee of our institute (Binzhou medical university) complied with the guidelines of the responsible government agency and with international standards (NIH publications No 80-23) revised 1996. Care was taken to reduce the suffering of the animals during the experiment. Rats (n = 45) were divided into three experimental groups each consisting of 15 individuals. Group I (RA) which served as control was exposed to room air (RA) while the remaining two groups (CIH, CIH + PCA) were exposed to CIH for 30 days (Fig. 1A).

### Dose response in pilot study

A pilot study was implemented taking 75 rats for establishing the optimal dose of the PCA. It was conducted by intraperitoneal injection with 0mg/kg (Normoxic), 0 mg/kg (30days Hypoxic), 5 mg/kg (30days Hypoxic), 15 mg/kg (30 days Hypoxic) and 45 mg/kg (30 days Hypoxic) to rats (n = 15 per group) for a duration of 7 days prior to exposure to CIH simulating. Following 28 days of CIH exposure, neurocognitive performance was observed by Step-through passive avoidance test later. There was no significant difference between the groups of 0 mg/kg and 5 mg/kg. No significant difference was observed between the groups of 15 mg/kg and 45 mg/kg doses but there was significant difference between 0 mg/kg (30days Hypoxic) group and 15 mg/kg group. Hence a daily dose of 15 mg/kg PCA was chosen.

### Drug administration

Rats in group III (CIH+PCA) received intraperitoneal injection of PCA at a daily dose of 15 mg/kg for 7 days prior to hypoxic exposure, and also during the whole exposure period of 30 days. All the animals that did not receive PCA were administered by intraperitoneal injection an equal volume of normal saline.

### Chronic intermittent hypoxia protocol

The chronic intermittent hypoxia model mimics the recurrent episodes of airway obstruction in OSA. Rats were respectively placed in specially designed chambers (60 × 40 × 25 cm) and exposed to intermittent hypoxia environment under the command of a gas control system (CYS-1 digital Hyp Oxyc system, Xinfei Analyzer Instrument Manufacture Inc., China). Based on well reported rodent models of obstructive sleep apnea^40^,^41^, a protocol was established (Fig. 1B). The alternating cycle lasted for 2 minutes consists of two phases. Firstly, O_2_ concentration could be decreased to a nadir level of 6.5–7% in 25–30 s by infusion of 99.99% nitrogen and sustained for 30–35 s. Then, by aeration of 99.50% O_2_ into the cabin during the next 10 s, the O_2_ concentration was guaranteed rising to 21% and lasted about 50 s. The CIH handling lasted 8 h (09:00–17:00) during the animals’ diurnal sleep period for 30 days. There was free access to food and water. The RA group was put in chamber continually aerated with room air and the O_2_ concentration was always kept at 21.0 ± 0.5%. Concentration of CO_2_, humidity and temperature inside the chamber were real-time monitored by detection electrode. The humidity and temperature in the chamber were maintained at 63 ± 3% and 21 ± 2 °C respectively. Concentration of CO_2_ in the chambers was kept at 0.03% by adjusting the ventilation. After CIH exposure, rats were returned to their cages. The behavior of the rats was closely observed.

### Behavioral tests

#### Step-through passive avoidance test

Step-through passive avoidance test was performed on the last two consecutive days of CIH period so as to examine memory consolidation. The apparatus consisted of two compartments (20 × 30 × 30 cm each) separated by a wall containing a door (8 cm in diameter) for connecting. The dark chamber was equipped with a grid floor and the other compartment was kept illuminated by a lamp. On the first day, each rat in three group (n = 15) was put in the illuminated chamber and left for 100 s for habituating the apparatus. Each rat was put individually in the illuminated chamber one hour later, and once it entered the dark chamber and all its four paws crossed, an electric shock (40 V, 0.5 A. 1 s) was applied to its feet through the floor grid. The animal was immediately returned to the illuminated compartment, or else it would be eliminated. 24 h later, the retention trials were conducted. Rats were placed in illuminated chamber again and the step through latency (STL) time between placement in illuminated compartment and entry to the dark one was recorded (maximum time allowed 300 s). If the rat still did not enter the dark chamber within 5 min, the test was stopped and the STL was recorded as 300 s. During these sessions, electric shock was not applied.

### Morris water maze

Morris water maze was conducted in three groups (n = 15) to study the effect of PCA administration on spatial reference learning and memory ability in the last 6 days of CIH exposure. The maze contains of a black rounded stainless steel tank of 200 cm diameter. The water was 35 cm deep and temperature was around 24–25 °C. The cistern was divided into four equal quadrants and four points as starting positions was established. An escape platform (10 cm in diameter, 2 cm below the water surface) was placed in a constant quadrant of the cistern. The rats were monitored by an overhead digital camera and computer assisted tracking system. In acquisition trials, rats had 4 trials per day separated by 20 min for 5 successive days. The starting position all rats entered were fixed. Rats were placed into water facing the wall of the cistern when a test began. The rat was allowed 120 s to find the platform, or else it was gently guided to it and allowed to stay on it for 30 s. Rats were dried with towel and electric hair dryer and returned to their cages after each trial. The pathlength in cm and latency in seconds to find the platform were recorded in each trial and mean pathlength and latency were calculated. On the 6th day, a probe trial was conducted. The rats were allowed to swim for 120 s and the platform was removed from original position at that time. The number of crossing over at the original platform position was recorded. The time (s) spent in all four quadrants including the target quadrant was measured too^42^.

### Tissue processing

Rats were deeply anesthetized with 10% chloral hydrate. Rats in three groups (n = 3) were perfused with 0.9% physiological saline via the left ventricle of the heart, and soon afterwards 200 ml of ice-cold 4% paraformaldehyde in 0.1 M phosphate buffer (PB), pH 7.4. The brain was divided into two halves along the midline. The prefrontal cortex and hippocampal CA1 region in one brain hemisphere were harvested rapidly. Upon that the specimens were fixed in 2.5% glutaraldehyde and post-fixed in 1% osmium tetroxide (Sigma, USA). Then they were processed for electron microscopy observation. The contralateral hemisphere was fixed for 24 h in 4% phosphate-buffered paraformaldehyde. Next, the tissue blocks were dehydrated in an ethyl alcohol series, cleared in xylene and then embedded in paraffin. The paraffin blocks were cut into 5 μm thick sections serially on a sliding microtome (Leica-RM2145, Germany). After that, the sections were then processed for immunohistochemistry and TUNEL assay. The rest of brain tissues of rats in each group (n = 12) were used for analysis of biochemical parameters as described below.

### Ultrastructure changes

The specimens were performed as described in detail above and then embedded with Epon812. The ultra-thin sections were cut using a LKB2088 vibratome and placed on single-hole grids. Then staining was conducted with 2% lead citrate (Merck, Germany) and 1% uranyl acetate (Merck, Germany). Ultrastructure changes of the cortex of the prefrontal lobe and hippocampus were examined by a transmission electron microscope (JEOL JEM-1400, Japan).

### DNA fragmentation by TUNEL assay

Apoptosis was assessed in 5 μm paraffin sections by detecting DNA fragmentation through TUNEL staining. TUNEL staining for apoptotic neurons in cortex of the prefrontal lobe and the hippocampus were performed in accordance with instructions of the manufacturer (Roche Corporation, Germany). The specimens in three group (n = 3) were observed using an upright microscope (OLYMPUS BX53F, Japan) and in at least six random fields of high-power field (×100). TUNEL positive cells were measured using software and the results showed the proportion of cells undergoing apoptosis.

### Oxidative stress and antioxidant status

Samples in three group (n = 8) were homogenized in 0.9% Saline and a 10% homogenate was obtain. The following is centrifuging at 10,000 rpm for 10 min. The clear supernatant was used for the further experiments. The activities of superoxide dismutase (SOD), glutathione peroxidase (GSH-PX), xanthine oxidase (XOD), NADPH oxidase (NOX), the concentrations of the malondialdehyde (MDA) and the protein in the supernatant were all measured according to the comercial detection kits. NOX detection kit was bought from solarbio and the others were purchased from Jiancheng Bioengineering (Nanjing, China). The activities of enzymes were expressed as units per milligram protein.

### Immunohistochemical staining

Immunostaining was performed according to a procedure routinely^18^,^43^. Two successive sections were collected every 50 μm and mounted onto polylysine-coated slides for detection of SYN and GFAP protein expression. After being deparaffinized and hydrated, the sections were subjected to antigen retrieval using a microwave for 30 min, and immersed in 3% hydrogen peroxide in methanol for 30 min to abolish endogenous peroxidase activity. Then the sections were washed in PBS and incubated with 5% normal goat serum to block nonspecific binding, followed by an overnight incubation with rabbit anti-SYN antibody (1:200, Abcam, MA, USA) and rabbit anti-GFAP antibody (1:50, Cell signaling, USA) at 4 °C. The sections were incubated with biotinylated goat anti-rabbit IgG for 2 h after washing. Afterwards, washing the paraffin sections (3 × 5 min) in PBS, and then colourated with 3,3-diaminobenzidin (DAB). The sections were kept in the dark at room temperature for 10 min. After washing slides (4 × 5 min) in PBS, counterstain was finished for 1 minute with hematoxylin in sections for SYN. The section for GFAP were processed with a Nissl staining (Baomanbio, Shanghai, China) and the sections were photographed. Slides were dehydrated with sequential ethanol washes of 1 minute each starting with 75%, followed by 80% and finishing with a 100% ethanol wash. Finaly slides were sealed with neutral gums. For determining the average values of the optical density (OD) of SYN and the Immunohistochemical index (IHC index is equal to the OD value multiplied by area) of GFAP, the image analysis system Image-Pro Plus 7.0 was used. Ten sections were analyzed for each rat and the average OD value was recorded.

### Western blotting

The hippocampus and prefrontal cortex were dissected from rat brains. Then the tissues (n = 3) were weighed and then homogenised in ice-cold RIPA buffer (Beyotime Institute of Biotechnology, China), after that samples were centrifuged at 10,000 rpm for 10 min at 4 °C. The protein concentration was measured by assay kits (BCA Protein Assay Reagent, Beyotime). Equal amounts of protein (100 μg) were boiled in sample buffer, separated by electrophoresis on 10–15% SDS-PAGE gels, and transferred to polyvinylidene fluoride membrane for 2 h at ice-bath. Membranes were blocked in 7% skim milk powder in TBS-T (TBS plus 0.5% Tween 20) at room temperature and then incubated overnight at 4 °C with primary antibody. Antibodies for Phospho-SAPK/JNK (Cell Signaling, 1:1000), SAPK/JNK (Cell Signaling, 1:1000), Phospho-P38(Cell Signaling, 1:1000), P38(Cell Signaling, 1:1000), ERK(Cell Signaling, 1:1000), Phospho-ERK(Cell Signaling, 1:2000), Bax (Cell Signaling, 1:1000), Bcl-2 (Cell Signaling, 1:1000), Cleaved Caspase-3 (Asp175) (Cell Signaling, 1:1000), BDNF (Santa Cruz, 1:500), pro-BDNF (sigma, 1:400), SYN (Sigma, 1:5000), c-fos (Bioworld, 1:1000)^44^,^45^, β-actin (Cell Signaling, 1:1000) antibody and anti-rabbit IgG HRP-linked secondary antibodies were employed. Proteins were visualized by enhanced chemiluminescence (ECL, Thermofisher, USA) on a FluorChem SP Imaging System. The intensities of the bands were quantified using scanning densitometry.

### Measurement of cytokine levels in the brain by ELISA

The brain tissues (n = 8) were homogenized in normal saline, then the homogenates were centrifuged at 4,000 rpm for 10 min and the supernatants were immediately separated by centrifugation and then divided into aliquots and stored at −80 °C. The concentration of IL-1β in the supernatants was determined using commercially available kit (Endogen, Woburn, MA, USA) specific for the cytokine according to the manufacturer’s instructions.

### Statistical analyses

The data of 5 day acquisition trials in Morris Water Maze were averaged for each session of four trials. The performance in trials was analyzed by using two-way Analysis of Variance (ANOVA). In the probe trial, time spent in target quadrant and time spent in each quadrant was analyzed using one-way ANOVA. The results of step-through passive avoidance test, histology and all biochemical estimations were performed by one-way ANOVA followed by a post hoc comparison test using LSD (equal variances assumed) or Dunnett’s T3 (equal variances not assumed) test to confirm the significant difference between the groups. For all statistical analysis values less than 5% (p < 0.05) were considered significant. Data are presented as mean ± SD unless otherwise stated.

## Additional Information

**How to cite this article**: Yin, X. *et al.* Protocatechuic acid ameliorates neurocognitive functions impairment induced by chronic intermittent hypoxia. *Sci. Rep.* **5**, 14507; doi: 10.1038/srep14507 (2015).

## Supplementary Material

Supplementary Information

## References

[b1] McNicholas W. T. & Bonsigore M. R. Management committee of EU COST ACTION B26. Sleep apnoea as an independent risk factor for cardiovascular disease: current evidence basic and research priorities. Eur. Respir. J. 29, 156–178 (2007). 17197482 10.1183/09031936.00027406

[b2] Mathieu A. *et al.* Effects of obstructive sleep apnea on cognitive function: a comparison between younger and older OSA patient. Sleep Med. 9, 112–120 (2007). 17513171 10.1016/j.sleep.2007.03.014

[b3] Xie H. *et al.* Brain-derived neurotrophic factor rescues and prevents chronic intermittent hypoxia-induced impairment of hippocampal long-term synaptic plasticity. Neurobiol. Dis. 40, 155–162 (2010). 20553872 10.1016/j.nbd.2010.05.020

[b4] Partinen M. & Telakivi T. Epidemiology of obstructive sleep apnea syndrome. Sleep 15, S1–S4 (1992) 1470800 10.1093/sleep/15.suppl_6.s1

[b5] Lumeng J. C. & Chervin R. D. Epidemiology of pediatric obstructive sleep apnea. Proc. Am. Thorac. Soc. 5, 242–252 (2008). 18250218 10.1513/pats.200708-135MG PMC2645255

[b6] Jackson M. L., Howard M. E. & Barnes M. Cognition and daytime functioning in sleep-related breathing disorders. Prog. Brain Res. 190, 53–68 (2011). 21531244 10.1016/B978-0-444-53817-8.00003-7

[b7] Gozal D. Sleep-disordered breathing and school performance in children. Pediatrics. 102, 616–620 (1998). 9738185 10.1542/peds.102.3.616

[b8] Gozal D. Effects of intermittent hypoxia on neurological function. In: Haddad G. G. & Yu S. P. , ed. Brain Hypoxia and Ischemia, 1st ed. Humana Press, NY, pp. 187–212 (2009).

[b9] Gozal D., Daniel J. M. & Dohanich G. P. Behavioral and anatomical correlates of chronic episodic hypoxia during sleep in the rat. J. Neurosci. 21, 2442–2250 (2001). 11264318 10.1523/JNEUROSCI.21-07-02442.2001 PMC6762394

[b10] Row B. W., Kheirandish L., Neville J. J. & Gozal D. Impaired spatial learning and hyperactivity in developing rats exposed to intermittent hypoxia. Pediatr. Res. 52, 449–453 (2002). 12193683 10.1203/00006450-200209000-00024

[b11] Tsai J. C. Neurological and neurobehavioral sequelae of obstructive sleep apnea. NeuroRehabilitation 26, 85–94 (2010). 20130357 10.3233/NRE-2010-0538

[b12] Row B. W. Intermittent hypoxia and cognitive function: implications from chronic animal models. Adv. Exp. Med. Biol. 618, 51–67 (2007). 18269188 10.1007/978-0-387-75434-5_5

[b13] Kaur C., Sivakumar V., Singh G., Singh J. & Ling E. A. Response of Purkinje neurons to hypobaric hypoxic exposure as shown by alteration in expression of glutamate receptors, nitric oxide synthases and calcium binding proteins. Neuroscience 135, 1217–1229 (2005). 16169666 10.1016/j.neuroscience.2005.06.023

[b14] Saligaut C., Chretien P., Daoust M., Moore N. & Boismare F. Dynamic characteristics of dopamine, norepinephrine and serotonin metabolism in axonal endings of the rat hypothalamus and striatum during hypoxia: a study using HPLC with electrochemical detection. Methods Find Exp. Clin. Pharmacol. 8, 343–349 (1986). 2426539

[b15] Partinen M. & Telakivi T. Epidemiology of obstructive sleep apnea syndrome. Sleep 15, S1–4 (1992). 1470800 10.1093/sleep/15.suppl_6.s1

[b16] Xu W. *et al.* Increased oxidative stress is associated with chronic intermittent hypoxia-mediated brain cortical neuronal cell apoptosis in a mouse model of sleep apnea. Neuroscience 126, 313–323 (2004). 15207349 10.1016/j.neuroscience.2004.03.055

[b17] Angelo M. F. *et al.* The proinflammatory RAGE/NF-κB pathway is involved in neuronal damage and reactive gliosis in a model of sleep apnea by intermittent hypoxia. PLoS One 9, e107901 (2014). 25265561 10.1371/journal.pone.0107901 PMC4180086

[b18] Burckhardt I. C. *et al.* Green tea catechin polyphenols attenuate behavioral and oxidative responses to intermittent hypoxia. Am. J. Respir. Crit. Care Med. 177, 1135–41 (2008). 18276944 10.1164/rccm.200701-110OC PMC2383994

[b19] Curran B. P., Murray H. J. & O’Connor J. J. A role for c-Jun N-terminal kinase in the inhibition of long-term potentiation by interleukin-1beta and long-term depression in the rat dentate gyrus *in vitro*. Neuroscience 118, 347–357 (2003). 12699771 10.1016/s0306-4522(02)00941-7

[b20] Thoenen H. The changing scene of neurotrophic factors. Trends Neurosci. 14, 165–170 (1991). 1713715 10.1016/0166-2236(91)90097-e

[b21] Bibel M. & Barde Y. A. Neurotrophins: key regulators of cell fate and cell shape in the vertebrate nervous system. Genes. Dev. 14, 2919–2937 (2000). 11114882 10.1101/gad.841400

[b22] Lewin G. R. & Barde Y. A. Physiology of the neurotrophins. Ann. Rev. Neurosci. 19, 289–317 (1996). 8833445 10.1146/annurev.ne.19.030196.001445

[b23] Xie H. & Yung W. Chronic intermittent hypoxia-induced deficits in synaptic plasticity and neurocognitive functions: a role for brain-derived neurotrophic factor. Acta Pharmacol. Sin. 33, 5–10 (2012). 22212429 10.1038/aps.2011.184 PMC4010262

[b24] Lin H. H., Chen J. H. & Wang C. J. Chemopreventive properties and molecular mechanisms of the bioactive compounds in Hibiscus sabdariffaLinne. Curr. Med. Chem. 18, 1245–1254 (2011). 21291361 10.2174/092986711795029663

[b25] Chen X. *et al.* Three-channel column-switching high-performance liquid chromatography with electrochemical detection for determining bioactive redox components in Salvia miltiorrhiza. J. Chromatogr. A 1256, 105–113 (2012). 22885041 10.1016/j.chroma.2012.07.061

[b26] Kakkar S. & Bais S. A. Review on Protocatechuic Acid and Its Pharmacological Potential. ISRN Pharmacol. 952943 (2014). 10.1155/2014/952943 PMC4005030 25006494

[b27] Tang X. L. *et al.* The cardioprotective effect of protocatechuic Acid on myocardial ischemia/reperfusion injury. J. Pharmacol. Sci. 125, 176–183 (2014). 25075424 10.1254/jphs.13247fp

[b28] Shi G. F., An L. J., Jiang B., Guan S. & Bao Y. M. Alpiniaprotocatechuic acid protects against oxidative damage *in vitro* and reduces oxidative stress *in vivo*. Neurosci. Lett. 403, 206–210 (2006). 16806694 10.1016/j.neulet.2006.02.057

[b29] Guan S., Jiang B., Bao Y. M. & An L. J. Protocatechuic acid suppresses MPP^+^-induced mitochondrial dysfunction and apoptotic cell death in PC12 cells. Food Chem. Toxicol. 44, 1659–1666 (2006). 16806628 10.1016/j.fct.2006.05.004

[b30] Parikh N. A. *et al.* Hypoxia-induced caspase-3 activation and DNA fragmentation in cortical neurons of newborn piglets: role of nitric oxide. Neurochem. Res. 28, 1351–1357 (2003). 12938857 10.1023/a:1024992214886

[b31] Gross A., McDonnell J. M. & Korsmeyer S. J. Bcl-2 family members and the mitochondria in apoptosis. Gene Dev. 13, 1899–1911 (1999). 10444588 10.1101/gad.13.15.1899

[b32] Li R. C. *et al.* Nitric oxide synthase and intermittent hypoxia-induced spatial learning deficits in the rat. Neurobiol. Dis. 17, 44–53 (2004). 15350964 10.1016/j.nbd.2004.05.006

[b33] Gozal E., Row B. W., Schurr A. & Gozal D. Development differences in cortical and hippocampal vulnerability to intermittent hypoxia in the rat. Neurosci. Lett. 305, 197–201 (2001). 11403939 10.1016/s0304-3940(01)01853-5

[b34] Gozal D. *et al.* Temporal aspects of spatial task performance during intermittent hypoxiain the rat: evidence for neurogenesis. Eur. J. Neurosci. 18, 2335–2342 (2003). 14622195 10.1046/j.1460-9568.2003.02947.x

[b35] Hota S. K., Barhwal K., Singh S. B., Sairam M. & Ilavazhagan G. Differential temporal response of hippocampus, cortex and cerebellum to hypobaric hypoxia: a biochemical approach. Neurochem. Int. 51, 384–390 (2007). 17531352 10.1016/j.neuint.2007.04.003

[b36] Sathesh Kumar S., Ravi Kumar B. & Krishna Mohan G. Hepatoprotective effect of Trichosanthes cucumerina Var cucumerina L. on carbon tetrachloride induced liver damage in rats. J. Ethnopharmacol. 123, 347–350 (2009). 19429383 10.1016/j.jep.2009.02.023

[b37] Nanduri J. *et al.* Xanthine oxidase mediates hypoxia-inducible factor-2α degradation by intermittent hypoxia. PLoS One 8, e75838 (2013). 24124516 10.1371/journal.pone.0075838 PMC3790816

[b38] Kim Y. J., Kim S. Y., Sung D. K., Chang Y. S. & Park W. S. Neuroprotective effects of L-carnitine against oxygen–glucose deprivation in rat primary cortical neurons. Korean J. Pediatr. 55, 238–248 (2012). 22844318 10.3345/kjp.2012.55.7.238 PMC3405156

[b39] Numakawa T. *et al.* Protective action of neurotrophic factors and estrogen against oxidative stress-mediated neurodegeneration. J. Toxicol. 405194 (2011). doi: 10.1155/2011/405194. 10.1155/2011/405194 PMC3135156 21776259

[b40] Chen L. *et al.* Oxidative stress and left ventricular function with chronic intermittent hypoxia in rats. Am. J. Respir. Crit. Care Med. 172, 915–920 (2005). 15976378 10.1164/rccm.200504-560OC

[b41] Drager L. F. *et al.* Chronic intermittent hypoxia induces atherosclerosis via activation of adipose angiopoietin-like 4. Am. J. Respir. Crit. Care Med. 188, 240–248 (2013). 23328524 10.1164/rccm.201209-1688OC PMC3778753

[b42] Netto C. A. *et al.* Effects of fetal hippocampal grafts onischemic-induced deficitsin spatial navigation in the water maze. Neuroscience 54, 69–72 (1993). 8515847 10.1016/0306-4522(93)90384-r

[b43] Li S. *et al.* Effects of dihydrotestosterone on synaptic plasticity of hippocampus in male SAMP8 mice. Exp. Gerontol. 48, 778–85 (2013). 23648585 10.1016/j.exger.2013.04.014

[b44] Zhang Q. W., Deng X. X., Sun X., Xu J. X. & Sun F. Y. Exercise promotes axon regeneration of newborn striatonigral and corticonigral projection neurons in rats after ischemic stroke. PLoS One 8, 11 (2013). 10.1371/journal.pone.0080139 PMC3833893 24260348

[b45] Srivastava P. *et al.* Unraveling the mechanism of neuroprotection of curcumin in arsenic induced cholinergic dysfunctions in rats. Toxicol. Appl. Pharmacol. 279, 428–40 (2014). 24952339 10.1016/j.taap.2014.06.006

