# Supplementary material for: Protocatechuic acid ameliorates neurocognitive functions impairment induced by chronic intermittent hypoxia
Source: Sci Rep. 2015 Sep 30;5:14507. doi: 10.1038/srep14507 (PMC4588513; doi:10.1038/srep14507)
Supplement: Supplementary Information [file srep14507-s1.pdf]

Protocatechuic acid ameliorates neurocognitive functions impairment induced by chronic intermittent hypoxia

Xue Yin[1,2@], Xiuli Zhang [1*@], Changjun Lv[2], Chunli Li[1,2], Yan Yu[1], Xiaozhi Wang[2], Fang Han[2*]

[1]*School of Pharmaceutical Sciences, Binzhou Medical University, Yantai, Shandong, 264003, China.*

[2]*Department of respiration, Binzhou Medical University Hospital, Binzhou, Shandong, 256603,*

[@]The first two authors are contributed equally to this work

Corresponding author: Xiuli Zhang

Tel：86-535-6913254

Fax：86-535-6913718

E-mail: zhangxiuli2008@163.com

Corresponding author: Fang Han

E-mail: hanfang1@hotmail.com

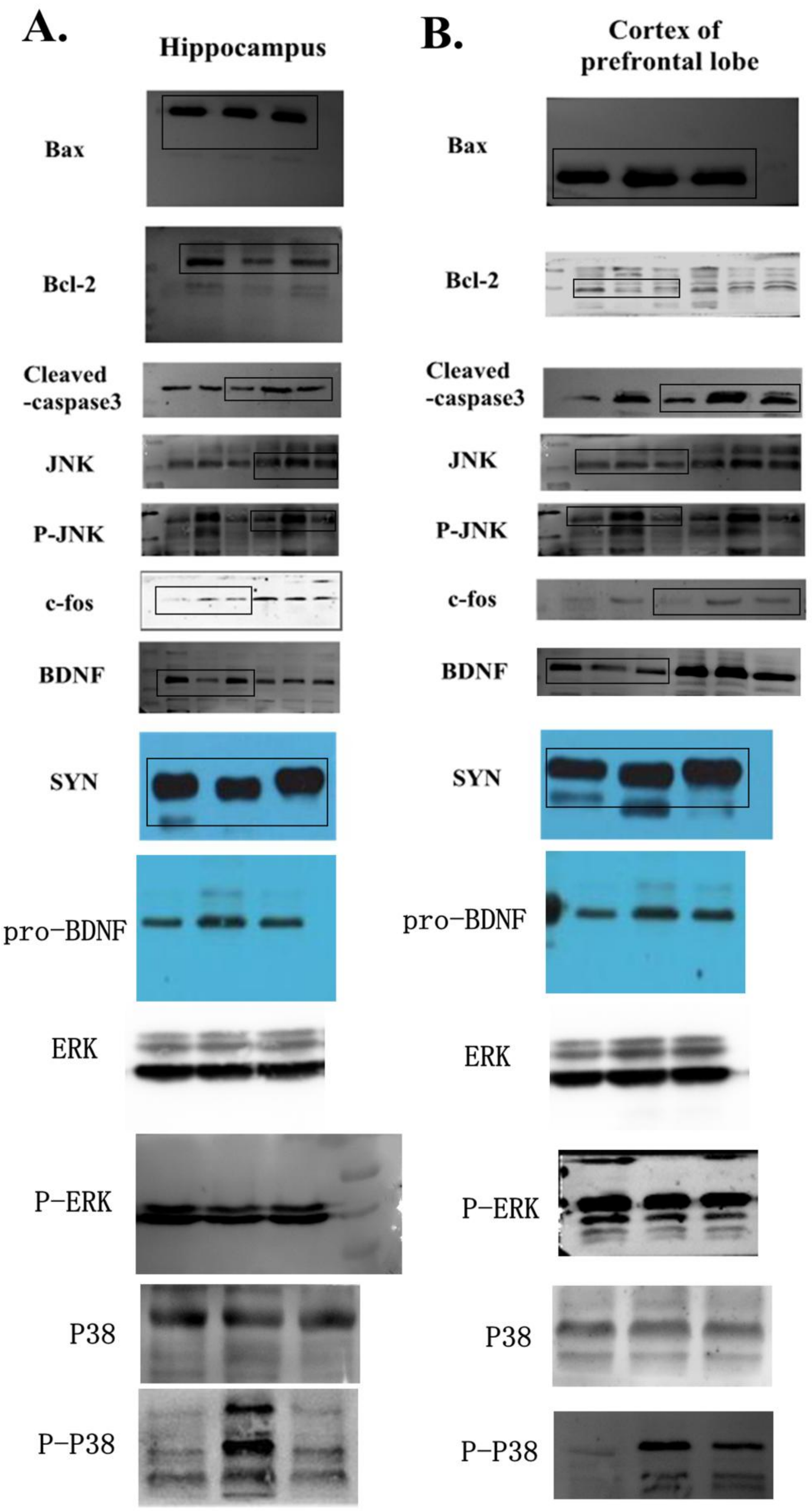
A.
Hippocampus
B.
Cortex of prefrontal lobe
Bax
Bcl-2
Cleaved -caspase3
JNK
P-JNK
c-fos
BDNF
SYN
pro-BDNF
ERK
P-ERK
P38
P-P38
